# Study on the influence law of cavities behind the shotcrete lining on the lattice girders

**DOI:** 10.1038/s41598-024-51191-8

**Published:** 2024-01-15

**Authors:** Chuande Qi, Junfeng Liu, Chao Zong, Weiliang Jiang, Weiteng Li, Yang Li, Jianquan Yan

**Affiliations:** 1 Shandong Provincial Key Laboratory of Civil Engineering Disaster Prevention, Shandong University of Science and Technology, Qingdao, 266590 China; 2 Qingdao Metro Group Co. Ltd, Qingdao, 266072 China; 3 Shandong Expressway Infrastructure Construction Co. Ltd, Jinan, 250000 China; 4 Zibo Traffic Construction Development Center, Zibo, 255000 China

**Keywords:** Engineering, Civil engineering

## Abstract

The field monitoring data showed that a small amount of main reinforcement bars of lattice girder at the arch of a tunnel were pulled, and the calculation showed that the initial support structure should be compressed. To find out the reason for the tension of the main reinforcement, the geological radar was used to detect the cavity in the sprayed concrete layer at the tension position. In order to clarify the tension mechanism of the main reinforcement and the influence of factors such as the position and size of the cavity on the main reinforcement, numerical simulations were carried out. The results show that the cavity causes the eccentric compression of the shotcrete layer, resulting in moment of the lattice girder and the change of the stress distribution of the main reinforcement. The main reinforcement experiences tensile stress when the cavity size surpasses 3 m × 0.2 m, reaching a tensile stress of 81 MPa at a cavity size of 6 m × 0.2 m. Notably, the cavity located at the foot of the arch is more likely to produce substantial tensile stress on the primary reinforcement compared to those at the arch crown and waist. The research results provide a theoretical basis for the interpretation and analysis of tunnel lattice girder monitoring data.

## Introduction

The support of tunnels usually adopts composite lining, where the initial support is usually a joint support system consisting of anchor, spray, steel mesh and lattice girder. The lattice girder and the surrounding rock through the concrete spray layer contact force transfer, in order to give full play to the role of support. However, the cavity behind the shotcrete is a common contact defect problem in tunnel engineering due to poorly compacted and solidified shotcrete during construction^1–4^, and the cavity will change the force condition of the lattice girder and adversely affect the safety and stability of the support structure^5–11^; Wang et al.^12^ found that the axial force of the lining structure at the boundary of the cavity increases significantly when there is a cavity behind the lining through numerical simulation; Zhang et al.^13^ found that the increase in cavity size leads to an overall reduction in the axial force of the tunnel structure; Zhang^14^ used numerical simulation to find that cavities contribute to the generation of circumferential cracks in the lining; Fang et al.^15^ found through model tests that the point contact between the lining and the surrounding rock near the cavity causes stress concentration in the lining; Lei et al. and Huang et al.^16,17^ used the extended finite element method based on the virtual crack model. Li et al. and Zhao et al.^18,19^ found that the cavity would lead to the formation of stress concentration in the arch at multiple locations and altering the stress state of the lining, which is not conducive to the performance of the arch bearing capacity by establishing the numerical model of the arch with cavity behind the lining. Ye et al.^20,21^ based on model experiments and numerical analysis, the study investigated the influence of cavity area and distribution on the force behavior of the lining structure. Zhang et al.^13,22,23^ evaluated and studied the mechanical performance of the lining structure under the influence of cavities by conducting model experiments on cavities of various positions, depths, and sizes behind the lining. The existing research on the cavity behind the support structure is mostly focused on the single-layer lining or the second lining in accordance with the lining, but there is less research on the force characteristics of the primary support structure, especially when the cavity exists behind the arch. In addition, most of the previous studies focused on the adverse effects of the cavity behind the support structure, while the field monitoring items were based on data such as arch axial force, spray layer strain and anchor axial force, and it was more difficult to link the monitoring data with possible tunnel diseases in actual engineering.

Based on the measured data of the initial support structure of a subway tunnel, this paper analyzes some difficult to explain axial tension data of the main reinforcement of the lattice girder, and found the correlation between the cavity and the tension of the main reinforcement of the lattice girder by using geological radar detection in the field, and then further carried out numerical simulation to study the mechanism and influence law of the cavity on the main reinforcement of the lattice girder.

## Project overview

### Engineering background

A line of Qingdao subway concealed excavation station buried depth 14–34 m, arch overburden thickness 13–27 m. The cave body is mainly located in slightly weathered rock and is mainly composed of granite, belongs to the category of relatively complete–complete, and relatively hard rocks–hard rocks, surrounding rock classification III_1_ grade–IV_2_ grade, arch III grade and IV grade accounted for 32% and 68% respectively, side wall III grade and IV grade accounted for 66% and 34% respectively, strength 55–90 MPa.

**Figure 1:**
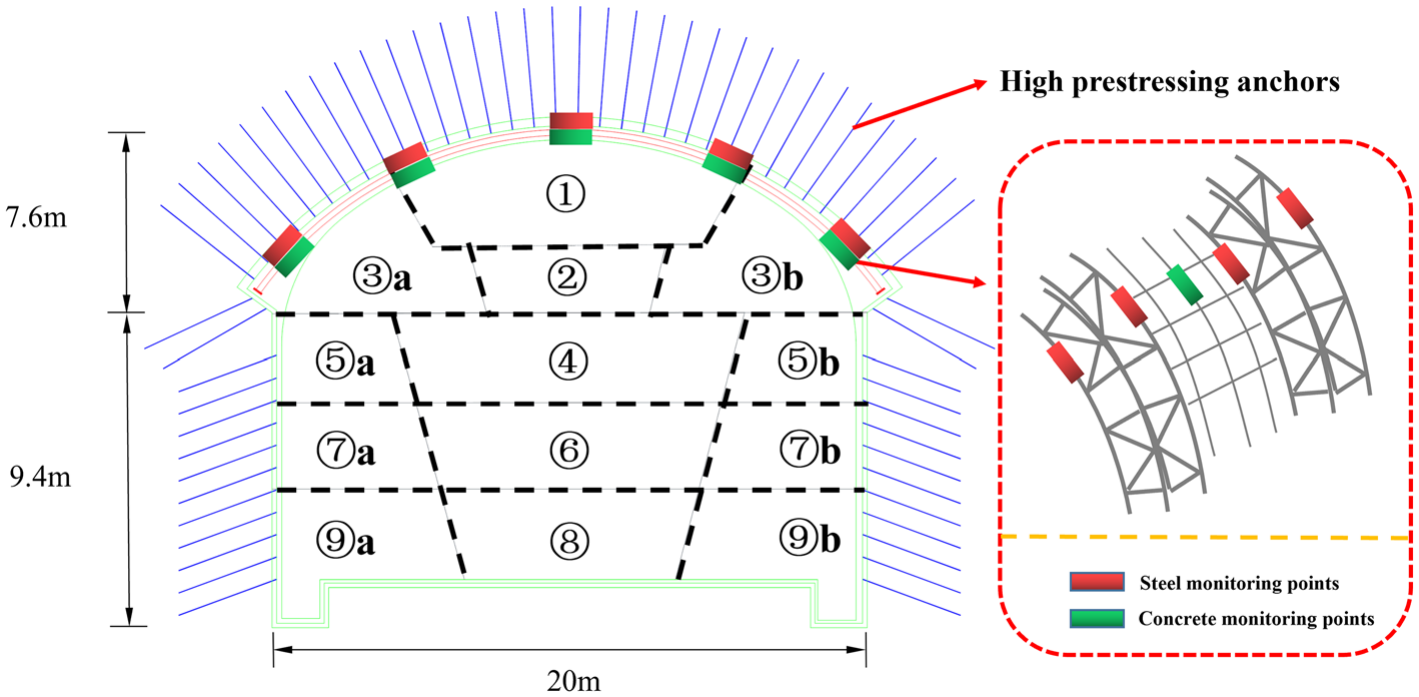
Construction sequence and monitoring point layout.

The tunnel adopts the form of three-centered round and straight sidewalls, and the cavern and support structure is designed according to the principle of the New Austrian Method, with the initial support system composed of anchor rods, shotcrete, main reinforcement network and lattice girder. The construction adopts the initial support arch cover method. Firstly, excavation of ① area is carried out, high prestressing anchors are driven and pretension is applied, reinforcement mesh is laid, four limbs of lattice girder are installed and welded to the reinforcement mesh, two sections of lattice girder are connected with each other by flanges, locking foot anchors are driven at the left and right ends of the lattice girder, followed by shotcrete. After advancing 15 m in ① area, excavation of ② and ③ area will start, and the support mode is the same as that of 1 area. After the arch is penetrated, the lower part of the excavation will be carried out sequentially, and the straight wall section will be supported by spraying anchors only, as shown in Fig. 1. The arch spacing is 1.2 m, 1 m and 0.8 m for III, IV_1_ and IV_2_ grade surrounding rock, respectively, and the anchors are arranged at intervals with the arch. The arch frame main reinforcement diameter is 22 mm, the anchor diameter is 18 mm, the length is 3.5 m, and the spray layer thickness is 35 cm.

### On-site monitoring

**Figure 2:**
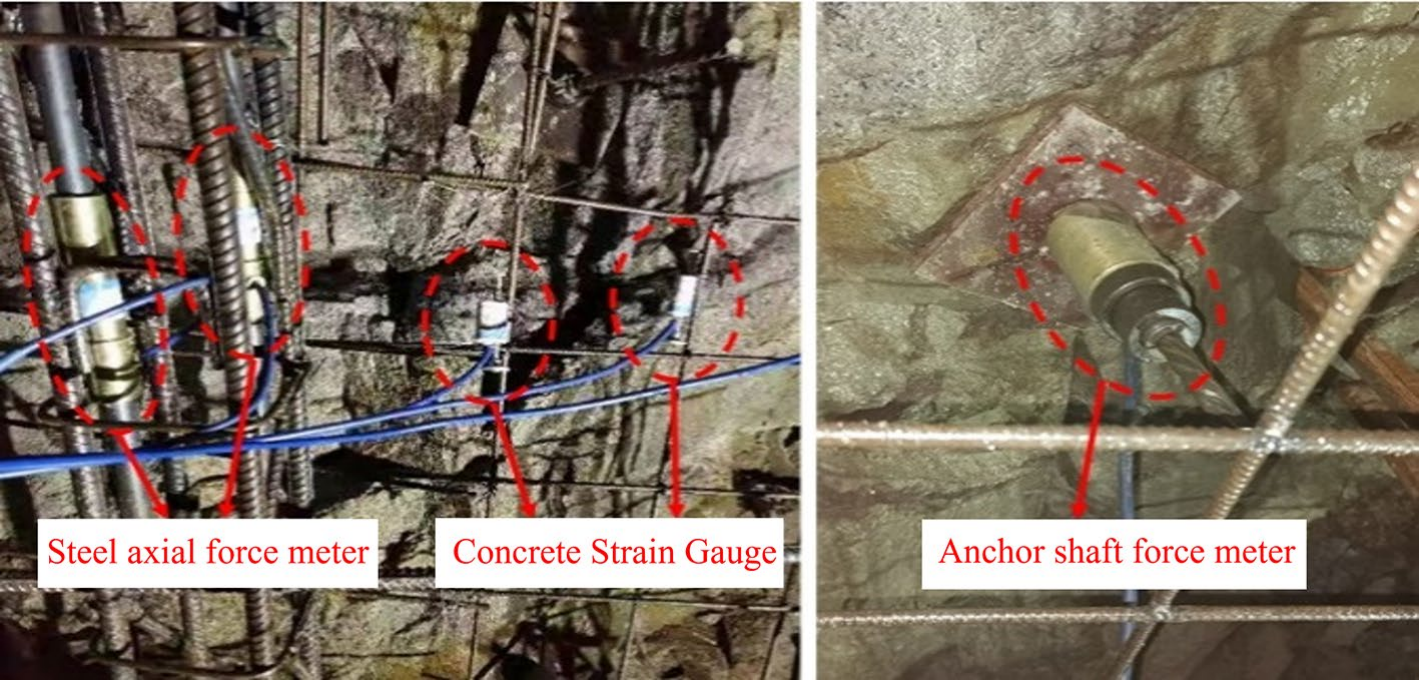
On-site installation of monitoring components.

The monitoring items include lattice girder main reinforcement axial force, concrete strain and anchor rod axial force. The monitoring points are the top of the arch, the waist of the arch and the location of the foot of the arch, in which two reinforcement axial force meters are installed at each monitoring point, and the four limbs of lattice girder are laid diagonally, as shown in Fig. 1. The monitoring elements use vibrating string type sensors, and the site is laid out as shown in Fig. 2.

Monitoring work collected the rebar meter indicates the frequency value, and the main reinforcement axial force value conversion is shown in Eq. (1),

1 $$F_{{\text{N}}} = \left( {f_{{\text{i}}}^{{2}} - f_{0}^{{2}} } \right)K$$

where F_N_ is the main reinforcement axial force; K is the conversion factor that comes with the equipment; f_i_ and f_0_ are the frequency value and the initial frequency value obtained from the ith monitoring, respectively, and i is the number of monitoring times, i = 1, 2, ……. The data measured before the installation of the equipment is set as the initial value, and each subsequent measurement represents the absolute value of the axial force of the main reinforcement of the lattice girder.

## Actual measurement data of the stress on the main reinforcement

### Overall pattern analysis

**Figure 3:**
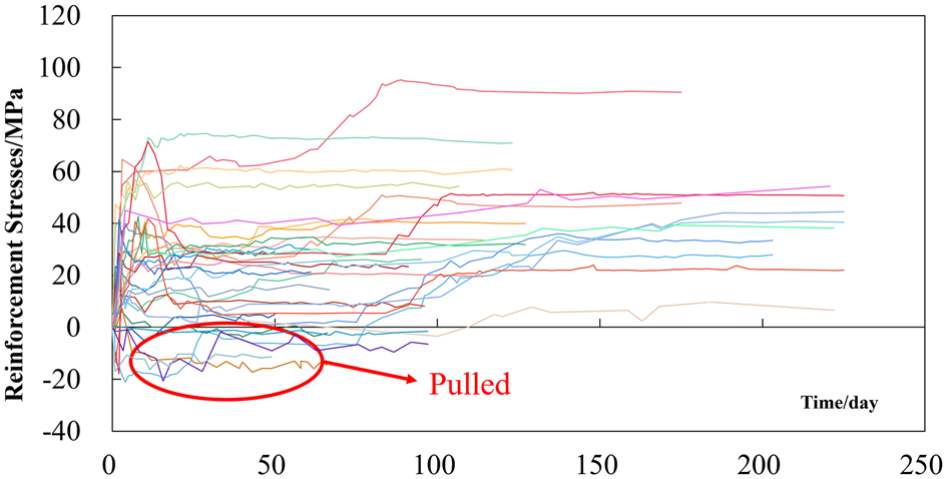
Stress monitoring curve of main reinforcement of lattice girder.

Figure 3 shows the measured stress data of the main reinforcement of lattice girder after tunnel arch penetration, where positive values represent compression and negative values represent tension.

It can be seen that: (1) The main reinforcement is primarily under compression, with a few instances of tension, accounting for 29.4% of the sections experiencing tension; (2) The curve is generally increasing–decreasing-stabilizing, and most of them are close to convergence after 20 days; (3) After stabilization, the stress is concentrated at 10–30 MPa, and much lower than the yield strength of HRB400 main reinforcement 400 MPa, still in the elastic stage.

### On-site testing

**Figure 4:**
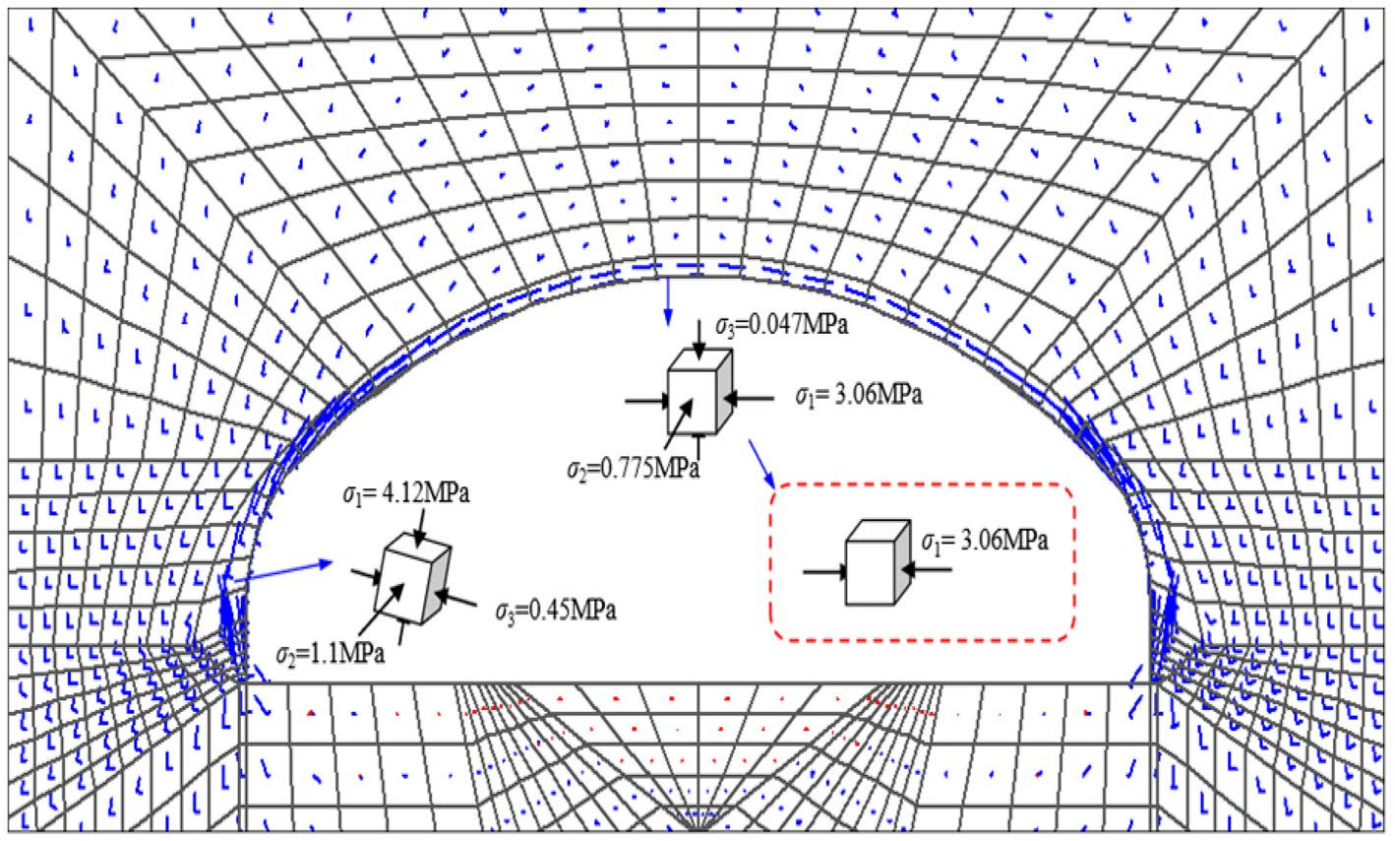
Principal stress tensor of surrounding rock and initial support structure of tunnel.

**Figure 5:**
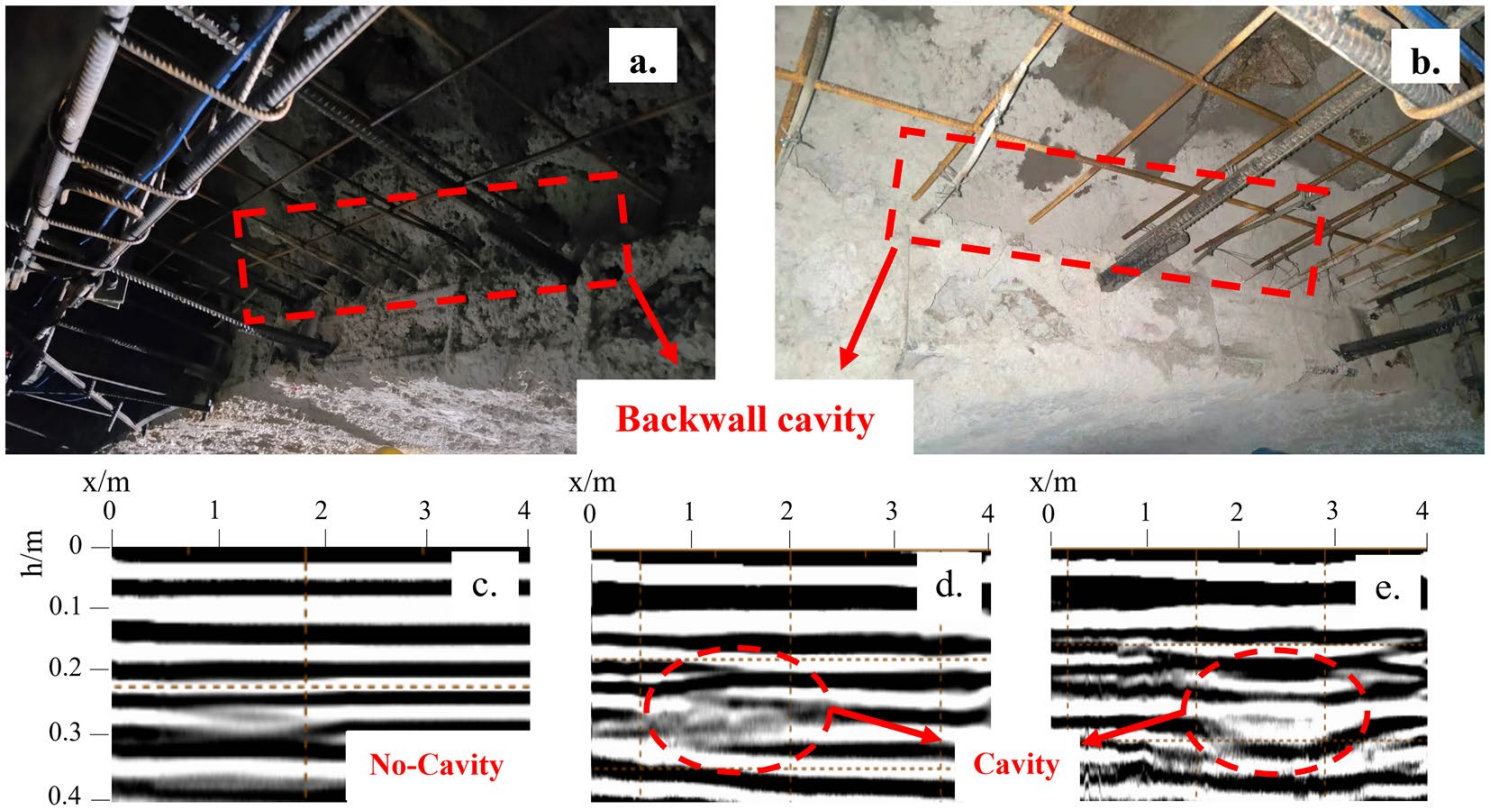
Detection image of cavities behind the shotcrete lining.

Figure 4 shows the tensor map of the principal stresses in the surrounding rock and initial support structure of the tunnel under ideal conditions. The model adopts the plane strain theory, with the surrounding rock and initial support structure using the Mohr–Coulomb constitutive model, and the anchor using the ideal elastic constitutive model. The numerical values and directions of the three principal stresses of the elements were obtained by solving through FLAC3D. It can be seen that the direction of the large main stresses in the concrete spray layer is basically distributed in the circular direction and are compressive stresses, the lattice girder main reinforcement appears tensile stresses do not meet the design expectations. However, during the construction process, it was observed that the presence of the lattice girder obstructed the concrete during the spraying process, resulting in insufficient thickness in certain areas that did not meet the design requirements, leading to the formation of local voids. as shown in Fig. 5a and b. Using ground penetrating radar to detect the tensile position of the main reinforcement on site, as shown in Fig. 5d and e, it can be seen from the comparison with the detection image without voids behind, Fig. 5c, that there are indeed voids inside the spray layer near the tensile main reinforcement. Therefore, it is inferred that there is a correlation between the cavities inside the spray layer and the tensioned main reinforcement of the lattice girder. In order to further verify this judgment and discuss the law of the influence of the cavities on the force of the main reinforcement of the grid, a numerical simulation study was carried out based on the field measurement results.

## Numerical simulation

### Simulation scheme

According to the research purpose, the numerical simulation scheme shown in Table 1 was designed, the schemes includes no-cavities, as well as cavities in the arch crown, arch waist, and arch foot, where the cavities length along the tunnel section in the circumferential direction are 2 m, 3 m, 4 m and 5 m, and the cavities thicknesses along the tunnel section in the radial direction are 0.1 m, 0.2 m and 0.3 m respectively.

**Table 1:** Simulation test plan.

Test name	Cavity location	Cavity length /m	Cavity thinkness /m
Control group without cavities	–	–	–
GD1–GD3	Vault	2	0.1, 0.2, 0.3
GD4–GD6		3	
GD7–GD9		4	
GY1–GY3	Arch waist	2	0.1, 0.2, 0.3
GY4–GY6		3	
GY7–GY9		4	
GJ1–GJ3	Arch foot	3	0.1, 0.2, 0.3
GJ4–GJ6		4	
GJ7–GJ9		5	

### Model and parameters

**Figure 6:**
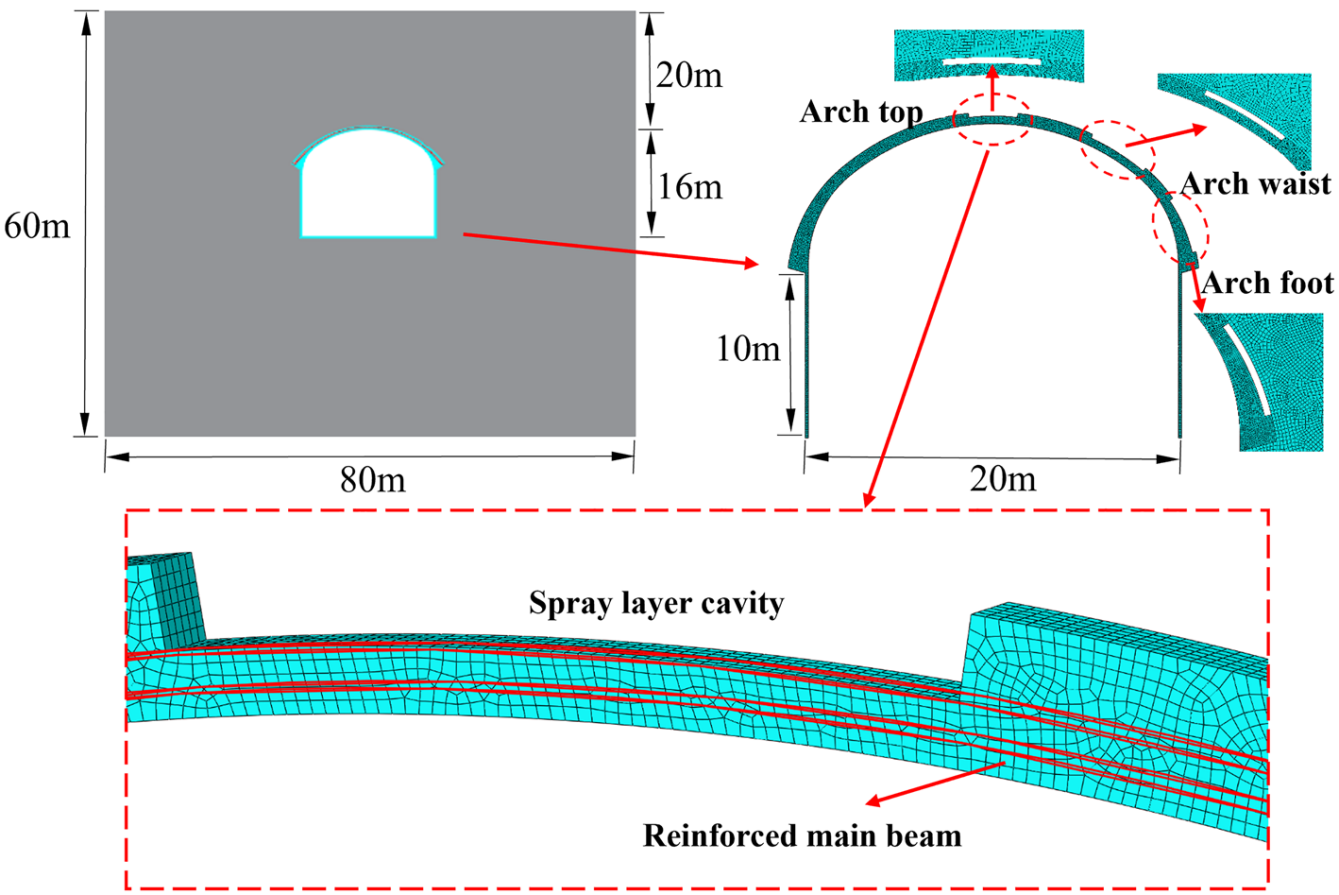
Numerical model of surrounding rock and initial support.

ABAQUS is used to establish the numerical model of the surrounding rock and primary support, and the model size and cavity location are shown in Fig. 6. Solid units are selected for concrete and surrounding rock, and beam units are used for the main reinforcement. The Moore-Coulomb model is used for the surrounding rock, the elastic–plastic model is selected for the main reinforcement, and the plastic damage model is used for the concrete. The surrounding rock is selected as IV_1_ level parameters, the main reinforcement of the lattice girder is HRB400 main reinforcement, the concrete type is C30, and the material parameters are shown in Table 2. The model is controlled by the method of normal displacement in the left, right, front and rear and the lower surface, and the overall load of the surrounding rock is derived from the self-weight of the surrounding rock and the support structure, and the initial ground stress is balanced first, and then the excavation support is balanced again.

**Table 2:** Material parameters.

`	Elastic modulus/GPa	Poisson's ratio	Heavy units /kN/m^3^	Compressive yield strength/MPa
Concrete	20	0.2	24.0	10
Surrounding rock	5	0.3	24.0	–
Rebar	200	0.25	78.5	400

### Result analysis

#### Mechanism analysis

**Figure 7:**
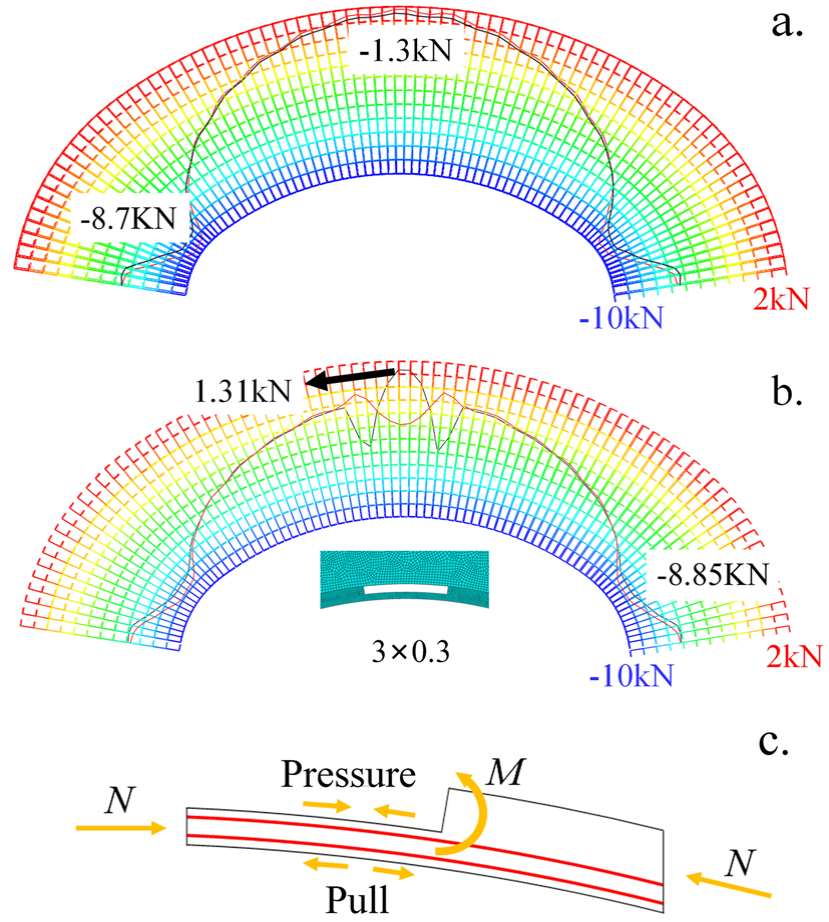
Axial force of main reinforcement with and without cavities and Tension mechanism.

**Figure 8:**
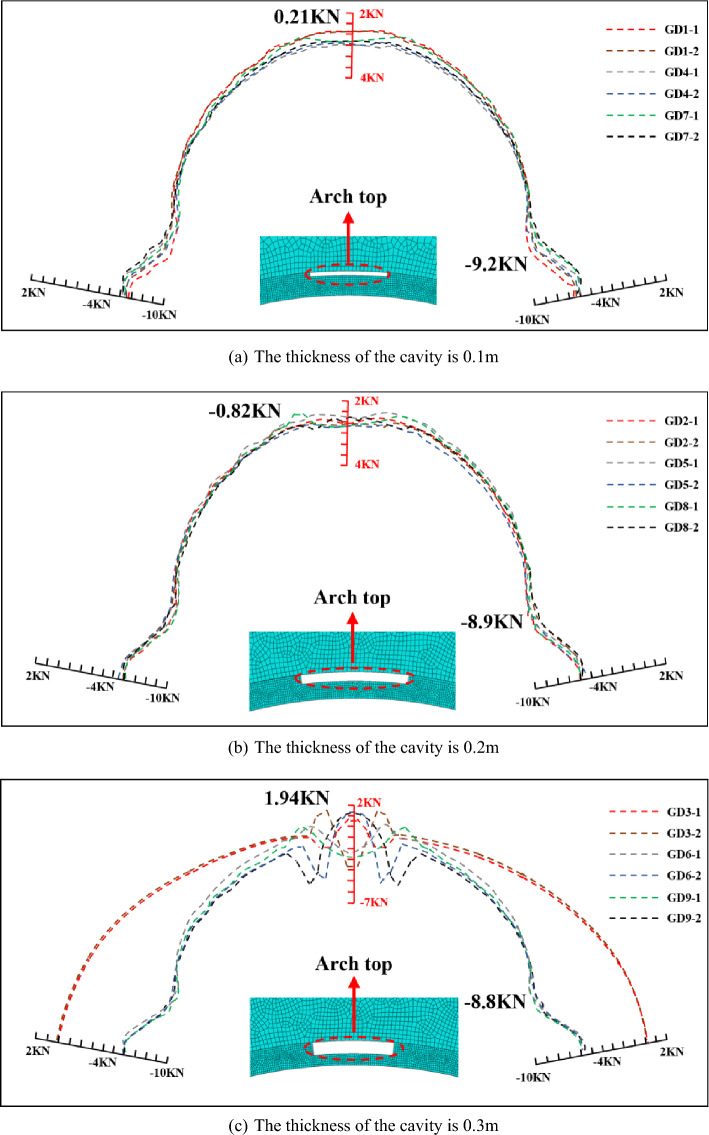
Axial force of main reinforcement with cavities of different sizes in the vault.

Figure 7a shows the axial force diagram of the main reinforcement in the no-cavity scheme, and Fig. 8b shows the axial force diagram of the main reinforcement when there is a 3 m × 0.3 m cavity at the top of the arch. The magnitude of the axial force is represented using a color scale, with colors ranging from blue to red, indicating stress values from compression to tension, with the ends corresponding to the peak compressive and tensile forces. The black curve represents the axial force of the main reinforcement on the surrounding rock side, while the red curve represents the axial force of the main reinforcement on the hollow side, in units of N. As can be seen from Fig. 8a, when there is no cavity, the main reinforcement is under pressure for the whole length, and the axial force at the foot of the arch is the largest, reaching 8.7 kN, the axial force at the top of the arch is 1.3 kN, and the axial force at the waist of the arch is between the foot of the arch and the top of the arch. The red part of the scale in Fig. 7b indicates the tensile force, and the main reinforcement at the top of the arch is under tension up to 1.3 kN, while all other positions are under pressure and the trend is similar to that when there is no cavity. In the middle and both ends of the cavity, the axial force curve of the main reinforcement on both sides of the cavity is inverse to each other and there is an axial force difference, which means that the lattice girder has generated a certain bending moment in the cavity area. Figure 7c shows the tensile mechanism of the main reinforcement at the cavity, because the concrete layer is missing a part here, the eccentric pressure has a bending trend, so that a certain local bending moment is generated, which leads to the main reinforcement under tension.

#### Cavities in the vault

Figure 8 shows the axial force of the main reinforcement when different sizes of cavities exist in the vault, the scale "test name-1" represents the axial force of the main reinforcement on the surrounding rock side, and the scale "test name-2" represents the axial force of the main reinforcement on the airside side, the positive value represents the tension, and the negative value represents the compression, the unit is KN. It can be seen the axial force of the main reinforcement near the cavities change obviously, with the increase of the length and thickness of the cavities, the axial force of the main reinforcement gradually changes from compression to tension. The increase of cavity thickness has a more significant effect on the main reinforcement axial force compared with the cavity length, and the presence of 0.3 m-thick cavity resulted in the main reinforcement exhibiting tension. Both sides of the main reinforcement axial force in the main reinforcement tensioned area had opposite trends and showed abrupt changes in axial force, the 2 m × 0.1 m and 2 m × 0.3 m cavities reduced the axial force of the arch waist and arch foot. Overall, the impact of the cavity in the arch vault has little impaction on the axial force of the main reinforcement, and a certain size cavity leads to the tension of the main reinforcement, but the value does not exceed 2KN, which is converted to tensile stress only 5.2 MPa, while the axial force of the main reinforcement in the area without cavity is similar to Fig. 7a, basically not affected by the cavity.

#### Cavity in the arch waist

**Figure 9:**
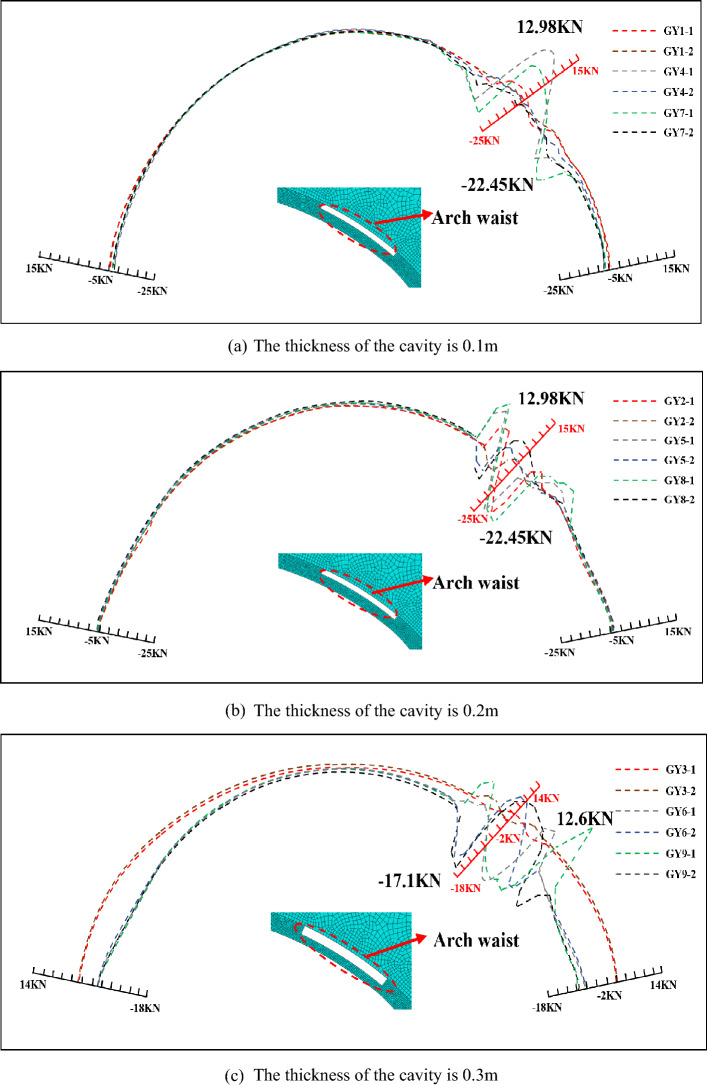
Axial force of main reinforcement with cavities of different sizes in the arch waist.

Figure 9 shows the axial force of the main reinforcement in the presence of cavity in the arch waist, it can be seen that the main reinforcement is under tension in all groups of tests of the cavity in the arch waist, compared with the top of the arch, the cavity in the arch waist position is more likely to lead to the main reinforcement under a significant tensile stress. The axial force of the main reinforcement at the cavity position changed obviously, and the axial force curves of the main reinforcement on both sides show a mutual inverse trend, and caused axial force difference, and the lattice girder had a certain bending moment here, that is, the cavity will cause the lattice girder to produce a certain bending moment. Most of the positive and negative axial forces of the main reinforcement are on the main reinforcement on the adjacent side, which means that the cavity behind the arch waist has more significant influence on the main reinforcement on the adjacent side. With the increase of cavity length, the maximum value of positive and negative axial force of main reinforcement increases, and when there is 0.2 m thick cavity behind the arch waist, the axial force of main reinforcement gets the maximum value of tension and compression. The cavity with 0.1 m thickness reduces the axial force value of the main reinforcement for the whole length, which does not exceed 1kN, and the same for 2 m × 0.3 m cavity. The maximum value of axial force appears in a group of 4 m × 0.2 m cavities in the arch waist, which is subjected to 12.98kN in tension, converting the compressive stress of the main reinforcement to 34 MPa, which is a small stress value for HRB400 main reinforcement, but the corresponding tensile strain is 0.00017, and the surrounding concrete has exceeded the cracking tensile strain of 0.0001.

#### Cavity in the arch foot

**Figure 10:**
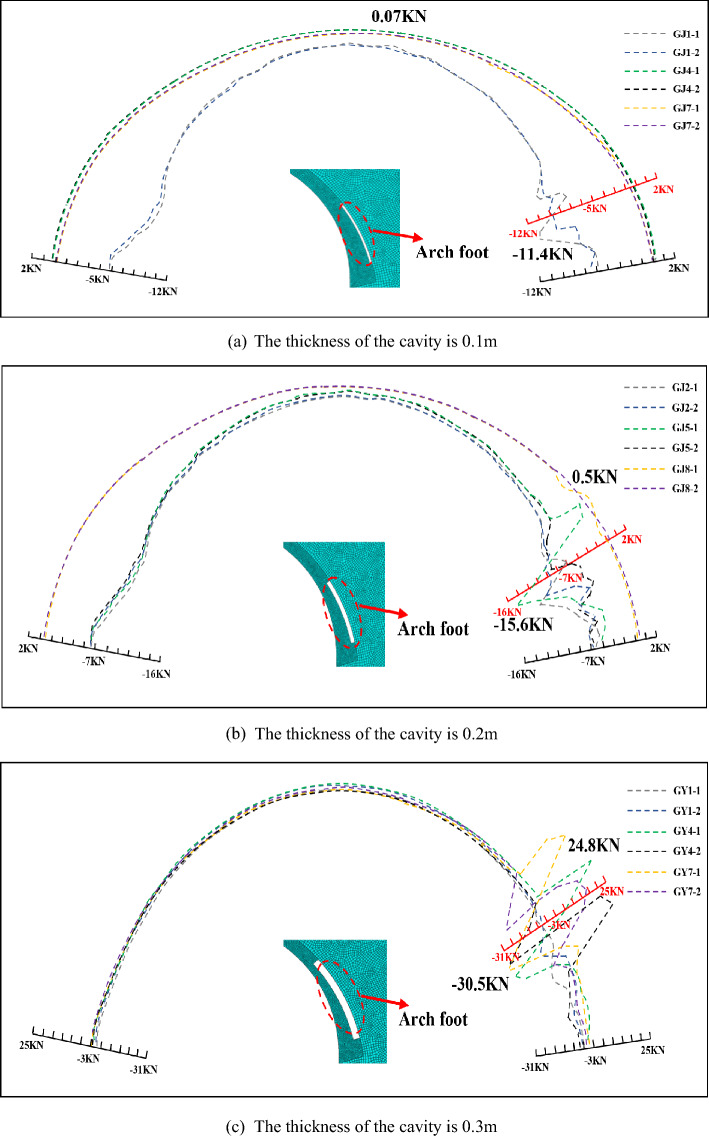
Axial force diagram of main reinforcement with cavities of different sizes in the arch foot.

Figure 10 shows the axial force of the main reinforcement when there is a cavity at the arch foot, it can be seen that compared with the top of the arch and the waist of the arch, the foot of the arch has to have a larger size cavity to cause the main reinforcement to be under tension, such as a 3 m × 0.3 m cavity. The most value of axial force is mostly located on the main reinforcement on the protruding side, which means that the main reinforcement on the protruding side is more affected by the cavity. The axial force curve of the main reinforcement at the cavity is mutually inverted and there are several axial force mutation points, 4 m × 0.3 m cavity has a large axial force difference between the main reinforcement on both sides, and the lattice girder has a certain bending moment at the cavity. The maximum value of tensile force is 24.76kN, and the converted tensile stress is 68 MPa, and the concrete immediately around the main reinforcement may have reached the cracking tensile strain.

#### Summary analysis

**Figure 11:**
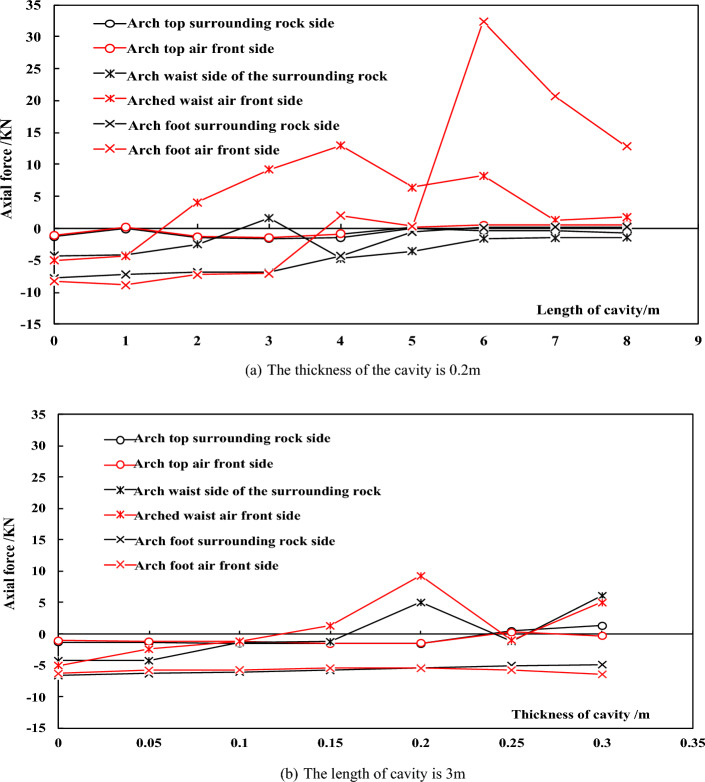
Axial force varies with cavities length and thickness.

The presence of cavities of the same size at different positions have different effects on the main reinforcement. Therefore, based on the test plan in Table 2, multiple sets of supplementary tests were designed for a length of 3 m and a thickness of 0.2 m. Figure 11a shows a cavity with a thickness of 0.2 m. As the length increases, the axial force of the main reinforcement on both sides changes. The maximum length of the cavity is taken as 8 m, with positive values representing tension and negative values representing compression. The values taken are the maximum axial force of the main reinforcement at the cavity. The axial force of the main reinforcement at each position first increases and then decreases, and cavities exceeding a certain size cause the main reinforcement to be tensioned. The inflection points of the curve in the figure mostly occur at a length of 4–6 m, indicating that when the length of the cavity is within this range, it is the most unfavorable situation for the main reinforcement. The axial force of the main reinforcement ranges from – 10 kN to 35 kN, which is in the elastic stage for HRB400 main reinforcement, especially when the arch cavity causes the main reinforcement to not exceed 2.3kN in tension, which is converted into a tensile stress of only 6 MPa. The cavity in arch waist causes a significant axial force difference between the main reinforcement on both sides, and there is a certain bending moment in the lattice girder at the cavity position. The size in the arch foot is 6 m × 0.2 m cavity generates a tensile force of 34kN on the main reinforcement, which is converted to a tensile stress of 81 MPa. The surrounding concrete reaches a tensile strain that has already cracked. Figure 11b shows the change in axial force of the main reinforcement on both sides of a 3 m long cavity with increasing thickness, and the maximum thickness of the cavity is taken as 0.3 m. The changes in the thickness and length of the cavity have a similar effect on the axial force of the main reinforcement, with the curves increasing first and then decreasing. The cavity behind the arch foot does not cause tension on the main reinforcement, indicating that the larger size of the cavity at the arch foot will have an impact on the axial force of the main reinforcement. The two figures show that the influence of the arch waist cavity on the axial force of the main reinforcement is more significant. When cavities of the same size appear at the arch waist, it is easy to cause the main reinforcement to be in tension, and the tensile value is also large; the larger size cavity at the arch foot will cause the main reinforcement to be tensioned; the influence of the arch cavity on the main reinforcement is relatively small.

## Conclusion

1. Field detection and monitoring show that there is a correlation between the cavity behind the shotcrete lining and the tension of the main reinforcement of the lattice girder, and the numerical simulation shows that the cavity generates a bending moment in the primary support structure, resulting in a significant change in the stress of the main reinforcement of the lattice girder, and the cavity will change the main reinforcement from compression to tension after it exceeds a certain size (3 m × 0.2 m).
2. The stress concentration will be generated at both ends of the cavity area, which is manifested by the sudden change of the axial force of the main reinforcement of the lattice girder, and the axial force curves of the reinforcement on both sides show a mutual inverse trend, and there is a certain bending moment of the lattice girder at this location.
3. The tensile stress of the tensile main reinforcement increases and then decreases with the increase of the length and thickness of the cavity. The cavity at the foot of the arch can produce considerable tensile stress in the main reinforcement, and the cavity of 6 m × 0.2 m can produce 81 MPa tensile stress in the main reinforcement, and the surrounding concrete has reached the cracking tensile strain; the cavity at the waist of the arch can produce tensile stress in the main reinforcement with a value smaller than that at the foot of the arch, and the cavity of 4 m × 0.2 m can produce 34 MPa tensile stress in the main reinforcement; the cavity at the top of the arch can produce tensile stress in the main reinforcement with a maximum of no more than 6 MPa.
